# Magnolin Protects against Contrast-Induced Nephropathy in Rats via Antioxidation and Antiapoptosis

**DOI:** 10.1155/2014/203458

**Published:** 2014-10-21

**Authors:** Feng Wang, Guangyuan Zhang, Yang Zhou, Dingkun Gui, Junhui Li, Tao Xing, Niansong Wang

**Affiliations:** ^1^Department of Nephrology, Shanghai Jiao Tong University Affiliated Sixth People's Hospital, 600 Yishan Road, Shanghai 200233, China; ^2^Department of Urology, Shanghai Jiao Tong University Affiliated First People's Hospital, 100 Haining Road, Shanghai 200080, China; ^3^Department of Nephrology, The First Affiliated Hospital of Harbin Medical University, No. 23 Youzheng Street, Nangang District, Harbin 150001, China; ^4^Florey Institute of Neuroscience and Mental Health, University of Melbourne, VIC 3010, Australia

## Abstract

*Background*. Magnolin is the major active ingredient of the herb *Magnolia fargesii* which has anti-inflammatory and antioxidative effects. Oxidative stress and apoptosis are involved in the pathogenesis of contrast-induced nephropathy (CIN). We hypothesize that Magnolin could protect against CIN through antioxidative and antiapoptotic properties. *Methods*. To test whether Magnolin could attenuate CIN, oxidative stress and apoptosis, *in vivo* and *in vitro*, we utilized a rat model of ioversol-induced CIN and a cell model of oxidative stress in which HK2 cells were treated with H_2_O_2_. Rats were assigned to 4 groups (*n* = 6 per group): control group, ioversol group (ioversol-induced CIN), vehicle group (CIN rats pretreated with vehicle), and Magnolin group (CIN rats pretreated with 1 mg/kg Magnolin). *Results*. The results showed that magnolin ameliorated the renal tubular necrosis, apoptosis, and the deterioration of renal function (*P* < 0.05). Furthermore, Magnolin reduced the renal oxidative stress, suppressed caspase-3 activity, and increased Bcl-2 expression *in vivo* and *in vitro*. *Conclusion*. Magnolin might protect CIN in rats through antioxidation and antiapoptosis.

## 1. Introduction

The use of contrast media is becoming more and more common in clinical diagnostic and interventional procedures [1, 2]. As a result, contrast-induced nephropathy (CIN) is a frequent complication in clinical medical practice. The incidence of CIN varies from 3% to 14% and can reach as high as 20% in patients at high risk [3–5]. Furthermore, CIN is the third leading cause of hospital-acquired acute kidney injury, accounting for ~12% of all cases of acute renal failure. CIN can result in an increased medical cost, long admission, disease deterioration, dialysis, and even death [6, 7]. Recent evidences suggest that CIN can lead to long-term decline in renal function as well as mortality [8, 9]. Thus, it is important and urgent to better understand the mechanism by which CIN occurs and to develop new preventive therapies.

The pathogenesis of CIN is primarily due to ischemia in the renal medulla resulting from intrarenal vasoconstriction, oxidative stress, and contrast media's renal toxicity. Oxidative stress plays a pivotal role in the pathophysiology of CIN which directly results in tubular damage, endothelial dysfunction, and dysfunction of tubular transport [10, 11]. Therefore, oxidative stress attenuation is a major goal in the CIN prevention research [12]. As an antioxidant,* N*-acetylcysteine is oxygen derived free radicals scavenger, which may lead to improved endothelial dysfunction [13, 14]. Apart from reducing the contrast media volume, cautiously avoiding hypovolemia, and carefully considering contrast delivery in high-risk patients, antioxidant is a promising strategy for the CIN prevention.

Magnolin is an important active ingredient of* Magnolia fargesii* volatile oil [15]. It was reported that* Magnolia fargesii* and Magnolin had antioxidative, anti-inflammatory, and vasodilatory effects [16]. But it is unknown whether Magnolin could protect against CIN. We hypothesized that Magnolin might protect against CIN in rats due to antioxidant and antiapoptotic effects. The aim of this study was to investigate whether Magnolin could prevent CIN and to identify the possible mechanisms.

## 2. Materials and Methods

### 2.1. Magnolin and Animals

The Magnolin reagents were provided by Dongfang Pharmaceutical Company (Shanghai, China). Before use, Magnolin was solubilized in DMSO for administration. Male Sprague-Dawley rats from Shanghai Science Academy animal center, weighing 200 ± 20 g, were housed in individual cages under controlled conditions of light (12 h dark/12 h light cycle) and 20~23°C. All the rats were allowed to have standard diet and tap water.

### 2.2. Rat CIN Model and Experimental Design

The rats were divided into control group (CTL) (*n* = 6), ioversol group (Iov) (*n* = 6), vehicle group (Veh) (*n* = 6), and Magnolin group (Mag) (*n* = 6). Rats in the Iov, Veh, and Mag groups were anesthetized with 50 mg/kg pentobarbital and given a tail vein injection of indomethacin (Sigma, USA) (10 mg/kg), followed by ioversol (Hengrui Corp., China) (3 g/kg organically bound iodine) and N-nitro-L-arginine methyl ester (L-NAME) (Sigma, USA). Rats in Mag group also receive a subcutaneous injection of Magnolin (1 mg/kg) 15 minutes prior to the CIN inducing injections. Rats in vehicle group received the same volume vehicle at each time point. The rats received recovery in metabolic cages for 24 hours for the urine sample collection. After 24 hours, the left kidney was harvested for associated measurements. The right kidney was fixed in 10% formalin for histological assessments. Blood samples were collected to isolate serum and then stored in a −80°C freezer.

### 2.3. Biochemical Markers Determination of Blood and Urine Samples

Automatic biochemical analyzer (Hitachi 7600, Ichige, Japan) was employed to determine blood urea nitrogen (BUN), serum creatinine (SCr). Two novel markers of early stage kidney injury, urinary kidney injury molecule-1 (uKIM-1), and serum neutrophil gelatinase-associated lipocalin (sNAGL) were measured using ELISA kits (R&D, USA).

### 2.4. Histological Examinations

The fixed left kidney was dehydrated in ethanol and embedded in paraffin. Kidney tissue blocks were cut into 3 *μ*m sections and subjected to hematoxylin-eosin (H.E.) staining and periodic acid-Schiff (PAS) staining. The histological scoring was assessed using grading tubular necrosis, loss of brush border, cast formation, and tubular dilatation in 10 randomly chosen, nonoverlapping fields. The renal injury degree was estimated by the following criteria: 0, none; 1, 0–10%; 2, 11–25%; 3, 26–45%; 4, 46–75%; and 5, 76–100%, as described previously [17].

### 2.5. Lipid Peroxidation/ROS Production of Renal Tissues

ROS level in kidney was assayed as previously described [18]. The nonpolar compound dihydrodichlorofluorescein diacetate (H2 DCFH-DA), after conversion to a polar derivative by intracellular esterases, can rapidly react with ROS to form the highly fluorescent compound dichlorofluorescein. The commercial available assay kit (E004, Nanjing Jiancheng Bioengineering Institute, Jiangsu, China) was used. As previously described [19], malondialdehyde (MDA) and superoxide dismutase (SOD) were detected in the kidney tissues. The content of MDA was determined by the thiobarbituric acid method (A003-1, Nanjing Jiancheng Bioengineering Institute, Jiangsu, China). The SOD activity assay kit (A001-1, Nanjing Jiancheng Bioengineering Institute, Jiangsu, China) was enrolled. SOD activity was determined by inhibition of nitroblue tetrazolium reduction due to superoxide anion generation by a xanthine-xanthine oxidase system. All the assays were carried out according to the manufacture's protocol using spectra microplate reader (model A-5082, Tecan, Australia).

### 2.6. Assessment of Renal Apoptosis in CIN Rats

A TUNEL staining for cell apoptosis in renal tissues was employed to evaluate the apoptosis extent following the instructions of the manufacturer (Roche Diagnostics, Mannheim, Germany).

### 2.7. Caspase-3 Activity and Bcl-2 Expression in Renal Tissues

The caspase-3 activity in renal tissues was measured using a commercial kit (Beyotime, Nantong, China). Western blot was employed to assess the Bcl-2 expression in renal tissues with an anti-Bcl-2 polyclonal antibody (#sc-492, Santa Cruz, USA) (dilution 1 : 200) as described previously [20].

### 2.8. An Oxidative Stress Model of HK2 Cells* In Vitro*

HK2 cells (ATCC, Manassas, Virginia) were cultured in K-SFM at 37°C 5% CO_2_, supplemented with 5 ng/mL human recombinant EGF and 0.05 *μ*g/mL bovine pituitary extract. HK2 cells were preconditioned with Magnolin (10 *μ*g/mL and 40 *μ*g/mL, resp.) followed by 500 *μ*mol/L H_2_O_2_ to induce oxidative stress injury. To investigate the antioxidative and antiapoptotic effects of Magnolin, caspase-3 activity (Beyotime, Nantong, China) and intracellular ROS (Cell Biolabs, San Diego, USA) were measured with commercial kits. In addition, Bcl-2 expression was determined using Western blot.

### 2.9. Statistical Analysis

The statistical software SPSS (Ver. 18.0) was used for data analysis. One-way ANOVA with Sidak post hoc test or Kruskal-Wallis with Dunn's post test was employed to determine the differences in groups. A value of *P* < 0.05 was considered significant.

## 3. Results

### 3.1. Magnolin Decreased Levels of SCr, BUN, sNGAL, and uKIM-1 in CIN Rats

**Figure 1:**
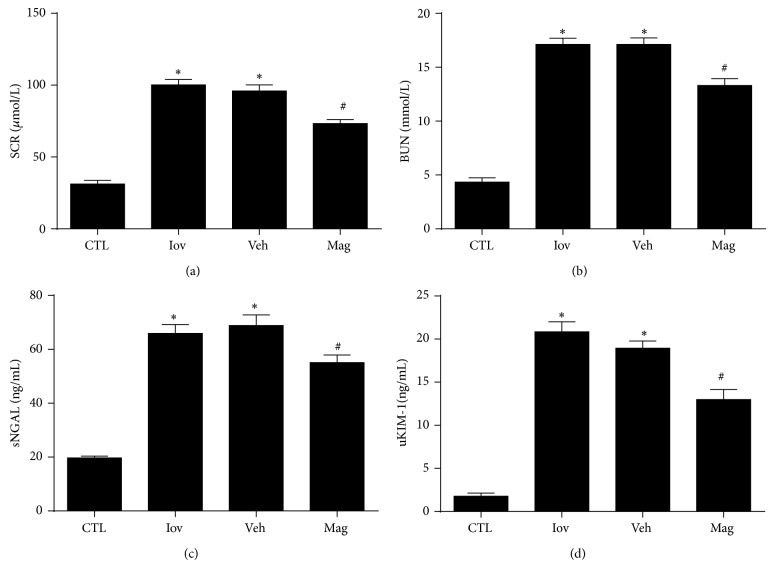
Magnolin decreased levels of serum creatinine (a), blood nitrogen urea (b), serum NGAL (c), and urinary KIM-1 (d) in CIN rats. ^*^ *P* < 0.01, versus CTL group. ^#^ *P* < 0.01, versus Veh group.

Animals subjected to CIN in Iov and Veh groups presented significantly increased levels of SCr, BUN, sNGAL, and uKIM-1 compared with CTL group. However, SCr, BUN, sNGAL, and uKIM-1 levels were all decreased in rats treated with Magnolin as shown in Figure 1. Thus, Magnolin can attenuate the levels of renal function parameters.

### 3.2. Magnolin Ameliorated Renal Histological Damage

**Figure 2:**
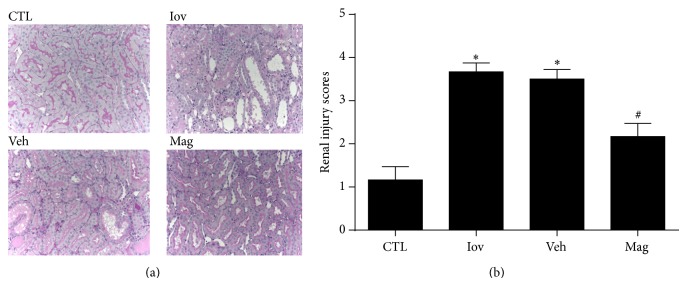
Magnolin ameliorated renal histological damage. (a) Representative renal sections from control, ioversol, vehicle, and Magnolin groups (PAS, 400x). (b) Renal injury scoring and quantitative analysis. ^*^ *P* < 0.01, versus CTL group. ^#^ *P* < 0.05, versus Veh group.

The tubular detachment, foamy degeneration, and necrosis can be seen in rats of Iov and Veh groups. Moreover, the most pronounced and severe alterations were observed in the corticomedullary boundary area. The morphological alterations in Iov and Veh groups were much more severe than that in Mag group. However, tubular scores in Mag group were ameliorated with the Magnolin treatment (Figure 2), which was consistent with Figure 1.

### 3.3. Magnolin Reduced Oxidative Stress in Renal Tissues

Elevated renal MDA and decreased renal SOD were observed in Iov and Veh groups compared with CTL group (*P* < 0.01) (Figures 5(a) and 5(b)). However, pretreatment with Magnolin reduced renal MDA levels and increased renal SOD levels dramatically. Thus, the results demonstrated that Magnolin might reduce oxidative stress from both directions in CIN rats.

### 3.4. Magnolin Inhibited Renal Apoptosis

**Figure 3:**
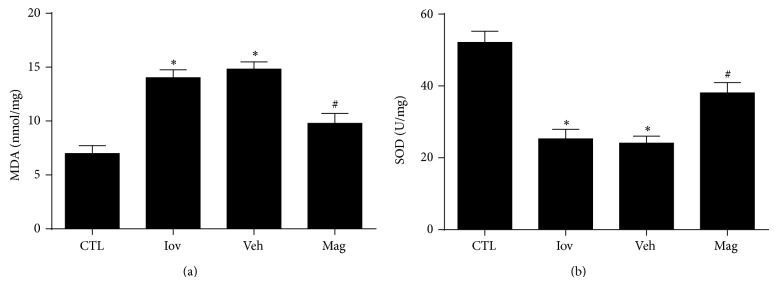
Magnolin reduced oxidative stress in renal tissues. (a) Renal MDA levels in rats from control, ioversol, vehicle, and Magnolin groups. (b) Renal SOD levels in rats from control, ioversol, vehicle, and Magnolin groups. ^*^ *P* < 0.01, versus CTL group. ^#^ *P* < 0.05, versus Veh group.

TUNEL assay revealed that the TUNEL-positive cells were much less in Mag group than that in Iov and Veh groups as shown in Figure 3. In comparison, Magnolin preconditioning reduced the tubular apoptosis in rats with ioversol-induced nephropathy.

### 3.5. Magnolin Decreased Renal Caspase-3 Activity and Increased Bcl-2 Expression in CIN

**Figure 4:**
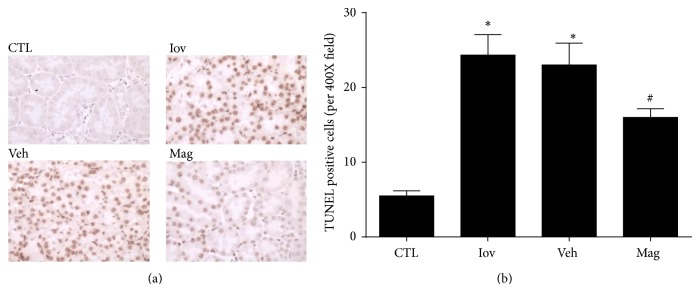
Magnolin inhibited renal apoptosis. (a) Representative renal sections from control, ioversol, vehicle, and Magnolin groups (TUNEL assay, 400x). (b) Quantitative analysis of tubular cells apoptosis. ^*^ *P* < 0.01, versus CTL group. ^#^ *P* < 0.05, versus Veh group.

**Figure 5:**
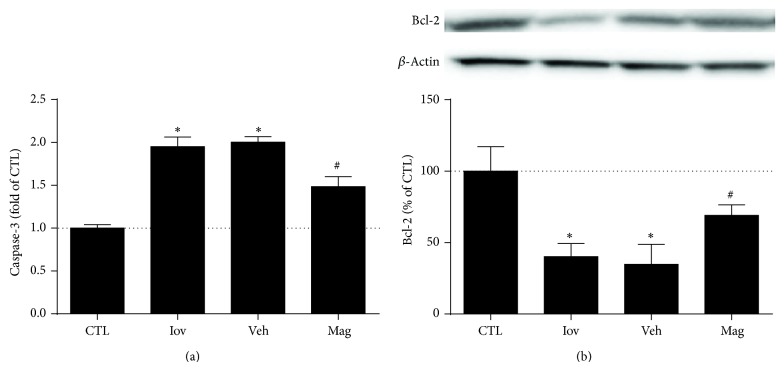
Magnolin decreased renal caspase-3 activity and increased Bcl-2 expression in CIN. (a) Renal caspase-3 activity in rats from control, ioversol, vehicle, and Magnolin groups. (b) Renal Bcl-2 expression (Western blot) in rats and quantitative analysis from control, ioversol, vehicle, and Magnolin groups. ^*^ *P* < 0.01, versus CTL group. ^#^ *P* < 0.05, versus Veh group.

To further determine whether Magnolin took protective effects on cell apoptosis, caspase-3 activity and Bcl-2 expression were measured. The results showed rats in Iov group and Veh group had significantly higher caspase-3 activity and lower Bcl-2 levels than that in CTL group (both *P* < 0.01). Furthermore, rats in Mag group exhibited decreased caspase-3 activity and increased Bcl-2 (*P* < 0.05) (Figure 4), which was consistent with Figure 3.

### 3.6. Magnolin Protected against Oxidative Stress and Apoptosis in HK2 Cells

**Figure 6:**
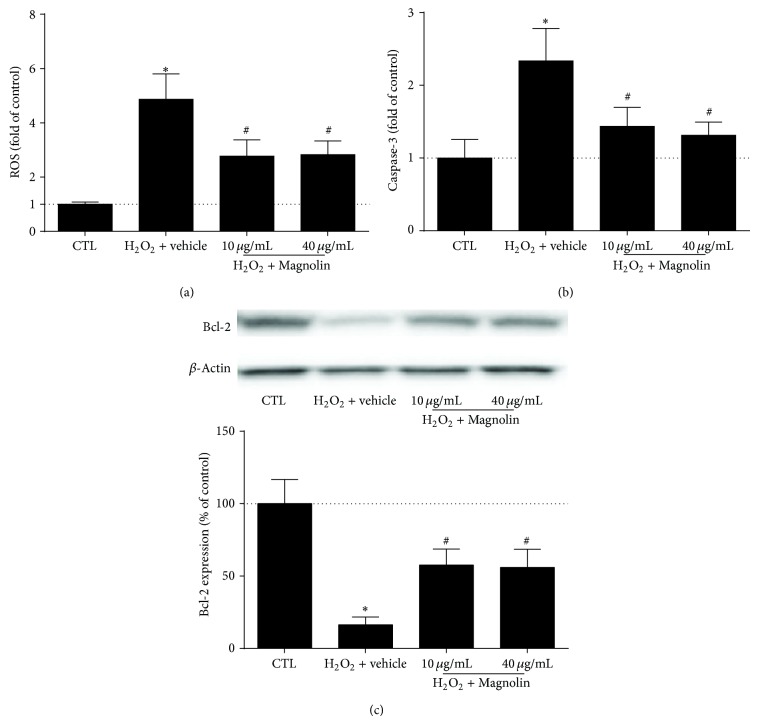
Magnolin protected against oxidative stress and apoptosis in HK2 cells. (a) The effects of Magnolin on ROS levels in HK2 cells treated with H_2_O_2_ (500 *μ*mol/L). (b) The effects of Magnolin on caspase-3 activity in HK2 cells treated with H_2_O_2_ (500 *μ*mol/L). (c) The effects of Magnolin on Bcl-2 expression in HK2 cells treated with H_2_O_2_ (500 *μ*mol/L). ^*^ *P* < 0.01, versus control group. ^#^ *P* < 0.05, versus H_2_O_2_ group.

The effects of Magnolin on ROS levels, caspase-3 activity, and Bcl-2 expression were examined in HK2 cells treated with H_2_O_2_. In contrast with control group, the ROS and caspase-3 levels elevated in HK2 cells treated with H_2_O_2_ (500 *μ*mol/L), while Bcl-2 expression was attenuated (*P* < 0.01). However, Magnolin (both 10 *μ*g/mL and 40 *μ*g/mL) decreased the ROS levels and caspase-3 activity and increased Bcl-2 expression (Figure 6).

## 4. Discussion

There are lots of circumstances in which it is medically necessary to utilize contrast-enhanced imaging, despite a significant risk of CIN. Thus, it is urgent to find the most beneficial treatments to prevent CIN from developing [12]. The present study demonstrated for the first time that Magnolin pretreatment before the administration of contrast media might prevent the acute kidney injury in rats. CIN is a renal injury not only due to the toxicity of contrast media, but also due to renal ischemia, oxidative stress injury, and cell apoptosis [11]. According to our results, Magnolin ameliorated renal injury, tubular necrosis, and the deterioration of renal function. Furthermore, Magnolin reduced oxidative stress, suppressed caspase-3 activity, and increased Bcl-2 expression* in vivo* and* in vitro*.

Oxidative stress plays an important role in the CIN pathogenesis. The administration of contrast media elevates ROS levels that can cause lipid peroxidation. Development or identification of new drugs that can scavenge ROS has been a major focus for the prevention of CIN in high-risk patients. In this study, Magnolin preconditioned rats expressed lower renal MDA, higher renal SOD, and reduced kidney injury. In addition, Magnolin decreased ROS generation and caspase-3 activity, while it increased the Bcl-2 expression in an oxidative stress model* in vitro*. Therefore, Magnolin presented antioxidative effects in this CIN model, which was consistent with previous reports [21]. It is deduced that Magnolin protecting against kidney injury in CIN rats might be due to its antioxidative effects.

Apoptosis is another mechanism of acute kidney injury including CIN [22, 23]. Suppressing apoptosis pathways may attenuate pathological alterations in CIN. It has been reported that both mitochondrial and intrinsic apoptosis pathways participated in the tubular injuries of CIN [24]. Bcl-2 is an antiapoptotic protein that can inhibit caspase-3. Increased Bcl-2 levels and decreased caspase-3 activity might contribute to reduced apoptosis [25]. In present study, we observed severe histological injury and extensive apoptosis in the CIN kidney, consistent with previous results [25, 26]. Moreover, increased caspase-3 activity and decreased Bcl-2 expression were partly reversed by Magnolin in CIN rats or HK2 cells treated with H_2_O_2_. Thus, it can be speculated that Manolin's renoprotection in CIN rats might be associated with Bcl-2/caspase-3 antiapoptosis pathway.

Our previous data showed that Magnolin protected against diabetic nephropathy through anti-inflammatory and antiplatelet effects [27]. Combined with the present results, Magnolin exhibited antioxidative and anti-inflammatory effects in rat kidney injury models, which was consistent with previous reports [28]. However, the detailed molecular mechanism that drives this protection remains unknown. Further studies focused on the molecular mechanisms will be important for both understanding the pathogenesis of CIN and evaluating Magnolin's therapeutic potential.

In conclusion, our data suggested that Magnolin exhibits renal protection in CIN model via antioxidative and antiapoptotic manners. Magnolin could be a novel antioxidant to the CIN therapy in the future.
